# Selectivity of the collagen-binding integrin inhibitors, TC-I-15 and obtustatin

**DOI:** 10.1016/j.taap.2021.115669

**Published:** 2021-10-01

**Authors:** Emma J. Hunter, Samir W. Hamaia, Donald Gullberg, Jean-Daniel Malcor, Richard W. Farndale

**Affiliations:** a Department of Biochemistry, University of Cambridge, Downing Site, Cambridge CB2 1QW, UK; b Department of Biomedicine, University of Bergen, Jonas Lies vei 91, N-5009 Bergen, Norway

**Keywords:** Integrin, Collagen peptides, Obtustatin, TC-I-15, Cell adhesion, C2C12

## Abstract

Integrins are a family of 24 adhesion receptors which are both widely-expressed and important in many pathophysiological cellular processes, from embryonic development to cancer metastasis. Hence, integrin inhibitors are valuable research tools which may have promising therapeutic uses. Here, we focus on the four collagen-binding integrins α1β1, α2β1, α10β1 and α11β1. TC-I-15 is a small molecule inhibitor of α2β1 that inhibits platelet adhesion to collagen and thrombus deposition, and obtustatin is an α1β1-specific disintegrin that inhibits angiogenesis. Both inhibitors were applied in cellular adhesion studies, using synthetic collagen peptide coatings with selective affinity for the different collagen-binding integrins and testing the adhesion of C2C12 cells transfected with each. Obtustatin was found to be specific for α1β1, as described, whereas TC-I-15 is shown to be non-specific, since it inhibits both α1β1 and α11β1 as well as α2β1. TC-I-15 was 100-fold more potent against α2β1 binding to a lower-affinity collagen peptide, suggestive of a competitive mechanism. These results caution against the use of integrin inhibitors in a therapeutic or research setting without testing for cross-reactivity.

## Highlights

• TC-I-15 inhibits the collagen-binding integrin, α2β1, with IC_50_ < 30 μM on GFOGER.
• Inhibition seems competitive with high potency, IC_50_ < 1 μM, on low-affinity GLOGEN.
• TC-I-15 inhibits α1β1 with similar potency, α11β1 weakly, but not α10β1.
• The KTS disintegrin, obtustatin, is specific for α1β1.
• Both inhibitors act on integrins in situ, not on isolated α-subunit I-domains.

## Graphical abstract

**Figure:**
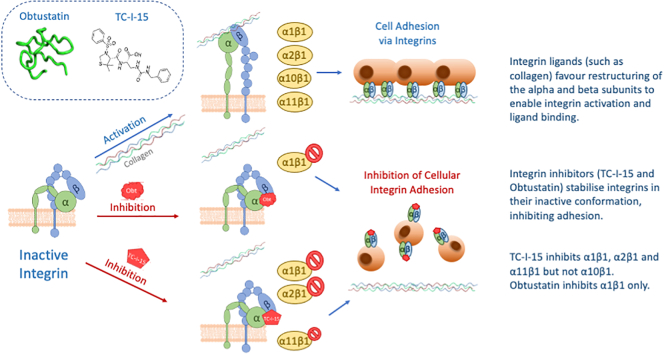

## 1 Introduction

Integrins are a family of glycoprotein transmembrane cell adhesion receptors that exist as α/β heterodimers. In humans, there are 18 α-subunits and 8 β-subunits that combine to form 24 different integrins (Arnaout et al., 2005; Barczyk et al., 2010; Hynes, 2002). Integrin expression is widespread but cell type-dependent, and most integrins bind a selection of extracellular matrix (ECM) components or cell-surface ligands. It is thought that cells express an excess of β-subunits and the expression of α-subunits determines surface receptor expression (Santala and Heino, 1991). They play essential roles in embryonic development, cell migration, proliferation and angiogenesis, and have been implicated in tumorigenesis and inflammation (Santala and Heino, 1991; Arnaout, 2016; van der Flier et al., 2010; San Antonio et al., 2009; Pozzi et al., 1998; Zhang et al., 2008; Senger et al., 2002; da Silva et al., 2010; Sottnik et al., 2013; Alique et al., 2014). Their primary function is to facilitate adhesion of cells to each other and to the ECM, but also to take part in matrix assembly (Musiime et al., 2021). Integrin-mediated signalling is essential for cell survival and many cell responses to growth factors are dependent on cell adhesion to a substrate via integrins (Barczyk et al., 2010; Meredith Jr. and Schwartz, 1997; Schwartz and Assoian, 2001; Byzova et al., 2000). As a consequence, many cell types must adhere to the matrix through integrins to survive (Schwartz and Assoian, 2001; Assoian, 1997).

Both the α and β subunits have a large extracellular N-terminal ‘head’ domain, a transmembrane domain and a small cytoplasmic C-terminal ‘tail’ (Arnaout et al., 2005; Barczyk et al., 2010; Hynes, 2002; Takada et al., 2007). By linking the ECM to the cytoskeleton, integrins mediate signal transduction from the environment, activating downstream signalling pathways such as focal adhesion kinases, talin and Src family kinases (Huveneers and Danen, 2009; Tadokoro et al., 2003; Calderwood et al., 1999). They form bi-directional signalling hubs that coordinate signals from several pathways (Arnaout et al., 2005; Giancotti and Ruoslahti, 1999) and form clusters with co-receptors and other integrins to amplify and modulate signals (Welf et al., 2012).

The four collagen-binding integrins, α1β1, α2β1, α10β1 and α11β1 all adhere to the GFOGER motif found primarily in collagens I and II, and to GLOGEN that is unique to collagen III. α2β1 and α11β1 adhere more strongly to GFOGER while α1β1 and α10β1 adhere more strongly to GLOGEN. Triple-helical peptides (THPs) containing these motifs have been synthesised and are used throughout this study (Emsley et al., 2000; Farndale, 2019; Knight et al., 2000; Zhang et al., 2003). Integrins α1β1 and α2β1 are widely expressed, whereas α10β1 is expressed primarily in chondrocytes (Barczyk et al., 2010; Takada et al., 2007) and α11β1 is expressed in fibroblasts, subsets of mesenchymal stem cells and subsets of cancer-associated fibroblasts (Zeltz and Gullberg, 2016; Popova et al., 2007; Popov et al., 2011; Shen et al., 2019; Zeltz et al., 2019; Zeltz et al., 2020). The range of pathways involving integrins highlights their importance in tissue function and homeostasis across a variety of organs. For example, whole organism ablation of β1 integrin in mice leads to embryonic fatal phenotype before E5.5 (Fassler and Meyer, 1995). Similar ablation of α1 or α2 subunits has a much less severe effect, with α1-null mice producing viable offspring with minor defects in collagen synthesis and angiogenesis (Gardner et al., 1996; Pozzi et al., 2000), whilst α2-null mice show delayed platelet aggregation (Chen et al., 2002). Both α1β1 and α2β1 enhance cancer cell migration and metastasis (Primac et al., 2019), for example, by upregulating matrix metalloproteinase synthesis via MAPK signalling (Ibaragi et al., 2011). Integrin α2β1 also promotes prostate cancer metastasis to the skeleton, resulting in a poor prognosis for patients (Sottnik et al., 2013). Unlike α1β1 and α2β1, which in tumours are expressed in both neoplastic and vascular cells, α11β1 is restricted to cancer-associated fibroblasts, and in a breast cancer model, restricts both tumour growth and metastasis (Primac et al., 2019). Understanding the inhibition of collagen-binding integrins is central to therapeutic targeting of integrins in these and other pathologies.

Nine of the integrins, including α1, α2, α10 and α11, contain an αI-domain, an insertion of about 200 amino acids in the β propeller structure of the α subunit head. The β subunits contain a similar βI-domain, and together, these I-domains regulate the activation status of the integrin (Arnaout et al., 2005; Shimaoka et al., 2002). The αI-domains determine ligand specificity despite their high homology; the rest of the subunit is more variable (Luo et al., 2007). The αI-domains adopt a Rossman fold with a five-stranded β-sheet surrounded by seven α-helices. Both the α and β I-domain contain a metal-ion-dependent adhesion site (MIDAS) that co-ordinates Mg^2+^ to which acidic residues, such as glutamate in the GFOGER motif, can bind. Conformational changes in the αI-domain are required to allow it to bind its ligand (Arnaout et al., 2005; Emsley et al., 2000; Shimaoka et al., 2002; Luo et al., 2007; Emsley et al., 1997). In the inactive conformation, the MIDAS is protected by a short C-helix. Upon integrin activation, the MIDAS re-organises and helix 7 translocates towards the α–β interface. This results in the relocation of the short C-helix onto helix 6, revealing the Mg^2+^ ion in a shallow groove that can accept the glutamate-containing motif from collagen or THPs (Emsley et al., 2000; Dickeson and Santoro, 1998; Hamaia et al., 2017). Communication occurs between residue E336, at the base of αI-domain helix 7, and the βI-domain MIDAS. The downward motion of helix 7 leads to reorganisation of the αI MIDAS and C-helix, and to activation of collagen-binding (Alonso et al., 2002; Yang et al., 2004).

Integrin inhibitors have therapeutic potential as well as being valuable research tools, and their study is therefore of great importance. The small molecule inhibitor, TC-I-15 (compound 15 in (Miller et al., 2009)) is considered to inhibit α2β1 by binding to the βI-domain MIDAS (Miller et al., 2009). The free carboxylate of the TC-I-15 dipeptide scaffold interacts with the MIDAS Mg^2+^ ion, alongside Y122 and N215, (ITB1_HUMAN Uniprot sequence) to block its ligation by E336 and so preventing the downward movement of helix 7. Consequently, the α subunit MIDAS remains concealed behind the C-helix and the integrin is stabilised in the inactive conformation. In platelets, this results in potent inhibition of platelet adhesion to collagen and the inhibition of subsequent thrombosis in animal models (Miller et al., 2009). TC-I-15 reportedly has no effect on the constitutively active E318A mutation of α2β1 (Miller et al., 2009). E318 is located at the top of helix 7, forming a salt bridge with R288 in the C-helix which must be broken to permit activation (Emsley et al., 2000). Thus, in E318A, helix 7 is decoupled from the upper surface of the αI domain which can reorganise independently of the βI-domain, and so E318A cannot be stabilised in the inactive conformation by TC-I-15.

Disintegrins are a family of integrin inhibitors derived from snake venoms (Arruda Macedo et al., 2015; Marcinkiewicz et al., 2003; Daidone et al., 2013). They are small cysteine-rich polypeptides, divided into several sub-groups based on their size or specificity. Disintegrins contain an integrin binding loop that, generally, competes directly with the natural ligand at its β-subunit binding site, and to fulfil this function, the integrin binding loop contains an RGD motif (Arruda Macedo et al., 2015). Similar motifs include MLD, VGD, KGD, and WGD, with each motif conferring a degree of integrin selectivity (see Table 1). Obtustatin, a KTS-disintegrin of just 41 residues, differs in its integrin-binding loop which contains W**KTS**LTSHY (Marcinkiewicz et al., 2003; Daidone et al., 2013), where the threonine residue (T22) is essential for α1β1 binding and the adjacent leucine (L24) contributes to high affinity (Kisiel et al., 2004). Two further KTS-disintegrins, viperistatin and lebestatin, and an RTS-containing disintegrin, jerdostatin, are also potent inhibitors of α1β1 (Kisiel et al., 2004), although where these disintegrins bind to α1β1 is not known. Obtustatin inhibits cellular, membrane-bound α1β1 and the isolated full length α1β1 but has no effect on recombinant αI-domains (Marcinkiewicz et al., 2003). The specificity of obtustatin, together with the absence of an acidic residue in its integrin-binding loop, suggests that obtustatin must interact with the α1 subunit, most likely as well as with the β1 subunit, close to the interface between the two. Obtustatin has been used to inhibit angiogenesis in vitro and in vivo in chick chorioallantoic membrane assays (Marcinkiewicz et al., 2003). Obtustatin also reduced tumour development by 50% in the mouse Lewis lung carcinoma model, and blocked melanoma growth in mice (Marcinkiewicz et al., 2003; Brown et al., 2008).

**Table 1:** A summary of the specificities of different motifs found on the integrin binding loop of various disintegrins. Adapted from (Arruda Macedo et al., 2015) and (Assumpcao et al., 2012)

Motif	Integrins targeted
RGD	α3β1, α4β1, α5β1, α6β1, α7β1, α8β1, αvβ1, αvβ3, αIIbβ3, and αvβ5
MLD	α3β1, α4β1, α5β1, α6β1, α7β1, α9β1, αIIbβ3, and α4β7
VGD/MGD	α5β1
KGD	αIIbβ3
WGD	α5β1, αVβ3 and αIIbβ3
KTS/RTS	α1β1

Neither of these two inhibitors, TC-I-15 and Obtustatin, has been tested on the more recently-characterised integrins, α10β1 or α11β1. Here, we tested their cross-reactivity with other collagen-binding integrins using recombinant integrin αI-domains, HT1080 cells that express only α2β1 and C2C12 cells that have been stably transfected to express only α1β1, α2β1, α10β1 or α11β1. A previously-characterised inhibitor of α2β1, monoclonal antibody 6F1, was also tested for comparison. The commercially-available small molecule α2β1 inhibitor, TC-I-15, was found to be non-specific as it also exerted an inhibitory effect on α1β1 and, at much higher concentrations, α11β1. Further, TC-I-15 was found to have no effect on α10β1 or purified α3β1. However, Obtustatin was found to inhibit only α1β1 of the four collagen-binding integrins. This highlights the importance of rigorously testing inhibitors for cross-reactivity before they can be used specifically.

## 2 Materials and methods

Integrin αI-domains were expressed as described (Siljander et al., 2004; Hamaia et al., 2012). TC-I-15 (4527/10), Obtustatin (4664/100 U) and α3β1 (2840-A3–050) were purchased from R&D technologies. Placental laminin-511 was purchased from Sigma L6274 (Wondimu et al., 2006). TC-I-15 was dissolved in NaOH (typically 220 μM) to obtain a final molar ratio of 1:1.1 (TC-I-15:NaOH). NaOH alone was used as a vehicle control for TC-I-15 whereas obtustatin and 6F1 were suspended in PBS. 6F1 was the generous gift of Dr. B. Coller, NY, USA.

### 2.1 Cell culture

C2C12s and HT1080s were grown in Lifetech DMEM/10% FBS and 1% penicillin/streptomycin. All cells were maintained under sterile conditions at 37 °C, 5% CO_2_. Cells were passaged using Trypsin/EDTA at 37 °C for 5 min and DMEM/10% FBS to quench the trypsin. C2C12 cells were transfected as described previously (Tiger et al., 2001). The C2C12-α10 clone was kindly made available by Dr. Evy Lundgren-Åkerlund, Xintela AB, Sweden.

### 2.2 Cellular static adhesion assays

Immulon 2HB 96-well plates were coated with 100 μl per well of peptides at 10 μg/ml in 0.01 M acetic acid overnight at 4 °C. After 3 × 200 μl/well PBS washes, plates were blocked with 200 μl/well filtered 3% BSA in PBS at RT for 1 h. Plates were washed, and 20,000 cells/well were added at room temperature (RT) for 1 h in serum-free media with 5 mM MgCl_2_ and varying concentrations of inhibitor. Unbound cells were washed away with 3× washes of 200 μl/well PBS. Adherent cells were lysed with 50 μl per well of 2% Triton X-100 in water for 1 h at RT. Cell number was quantified using 50 μl per well of the Roche cytotoxicity LDH kit. The catalyst and substrate solutions of the kit were mixed at a ratio of 1:45 and 50 μl was added to the wells to detect LDH in the cell lysate. A_490_ was read in a SpectraMax 190 microplate reader (Molecular Devices). Each condition was performed in triplicate, with at least three independent repeats.

### 2.3 Protein static adhesion assays

Immulon 2HB 96-well plates were coated with 100 μl per well of peptides at 10 μg/ml in 0.01 M acetic acid. After 3 × 200 μl/well washes with washing buffer (1 mg/ml BSA in TBS), plates were blocked with 200 μl per well of filtered 3% BSA in TBS for 1 h at RT. αI-domains were added at 10 μg/ml in washing buffer for 1 h at RT with 5 mM MgCl_2_ and various concentrations of inhibitor as stated. After 3 × 200 μl/well washes with washing buffer, anti-GST detection antibody (GE Healthcare HRP conjugated anti-GST GERPN1236) were added for 1 h. Plates were washed 4× with 200 μl per well of washing buffer and bound protein was quantified using the Pierce™ TMB Substrate Kit. The colorimetric reaction was stopped with an equal volume of 1 M H_2_SO_4_ and A_450_ was read as above. Each condition was performed in triplicate and each experiment was repeated at least three times using different preparations of proteins.

### 2.4 Collagen peptides

Peptides containing the sequences GFOGER or GLOGEN (single amino acid nomenclature where O is hydroxyproline) were synthesised as C-terminal amides, and assembled as triple-helical homotrimers, as described previously (Knight et al., 2000; Raynal et al., 2006). Peptides contain a binding motif (such as GFOGER) flanked on either side by five GPP repeats and a GPC triplet at both the N- and C-termini. Peptides were synthesised using solid phase Fmoc peptide chemistry on a CEM Liberty Blue microwave-assisted synthesiser. Peptides were then cleaved from the resin beads, purified by preparative reverse-phase high performance liquid chromatography, freeze dried and characterised by mass spectrometry. Peptides were dissolved at 5 mg/ml in 0.01 M acetic acid, heated to 70 °C for 5 min and allowed to cool overnight to enable triple helix folding. Peptides were diluted from this stock solution to 10 μg/ml in 0.01 M acetic acid for the coating of empty tissue culture wells prior to experiments. The peptide GPP10 (sequence: GPC(GPP)_10_GPC-amide) is used as a negative control. The GPP repeats adopt triple-helical structure but the sequence is inert, with no binding motifs present.

### 2.5 Statistical analysis

PRISM 8.2.1 or later (GraphPad, San Diego, CA, USA), was used for all statistical tests. For single concentration studies one-way ANOVA was used with Sidak's multiple comparisons test to compare means for each condition with the appropriate control. Means ± SD are shown in all data sets. Inhibition curves were analysed using nonlinear regression comparing each data set with either a horizontal line model (null hypothesis, no inhibition) or three-parameter dose-response curve (inhibition occurs). Three-parameter curves were constrained with lower value <0.1, reflecting inhibition of adhesion to baseline GPP10 values, and IC_50_ > 0.

## 3 Results

### 3.1 Integrin αI-domain adhesion to collagen peptides is unaffected by TC-I-15 and Obtustatin

**Fig. 1:**
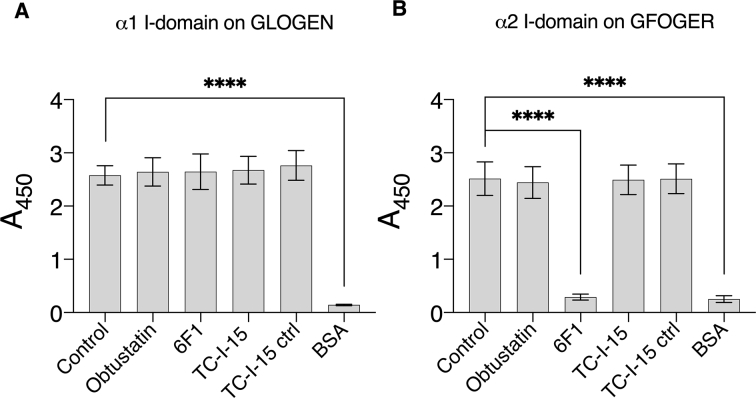
Inhibition of binding of recombinant αI-domains to peptide coatings. Adhesion of A, α1 I-domain to GLOGEN and B, α2 I-domain to GFOGER, was measured using anti-GST-HRP conjugated antibody as described in Materials and methods, and is shown as mean A_450_ ± SD. TC-I-15 (200 μM), Obtustatin (20 μM) or 6F1 (10 μg/ml) were used as indicated. “TC-I-15 ctrl” refers to the vehicle control, 220 μM NaOH. Each condition was performed in triplicate and repeated 3 times. **** denotes *P* < 0.0001.

To confirm that these inhibitors have no effect on isolated αI-domains, GST-tagged αI-domains were tested in static adhesion assays where plates had been coated with either GLOGEN (for the α1 I-domain) or GFOGER (for the α2 I-domain) in the presence or absence of the inhibitors obtustatin, 6F1 and TC-I-15. Previous work in this laboratory established that GFOGER is a strong ligand and GLOGEN a moderate ligand for α2β1 whereas GLOGEN is a strong ligand and GFOGER a moderate ligand for α1β1 (Farndale, 2019; Knight et al., 2000; Farndale et al., 2008). The α2β1 inhibitory antibody, 6F1, was also included for comparison (Coller et al., 1989). Fig. 1A and B show levels of adhesion after inhibition. Antibody 6F1 was a potent inhibitor of the α2 I-domain adhesion to GFOGER (control mean = 2.51 ± 0.3; 6F1 mean = 0.29 ± 0.056, *p* < 0.0001) but not the α1 I-domain adhesion to GLOGEN, confirming its specificity for α2β1. This confirms that, whereas 6F1 directly targets the free αI-domain, obtustatin and TC-I-15 are not able to do so, in agreement with the literature.

### 3.2 In C2C12 cells, TC-I-15 inhibits adhesion of α1β1, α2β1 and α11β1, but not α10β1, to collagen peptides

**Fig. 2:**
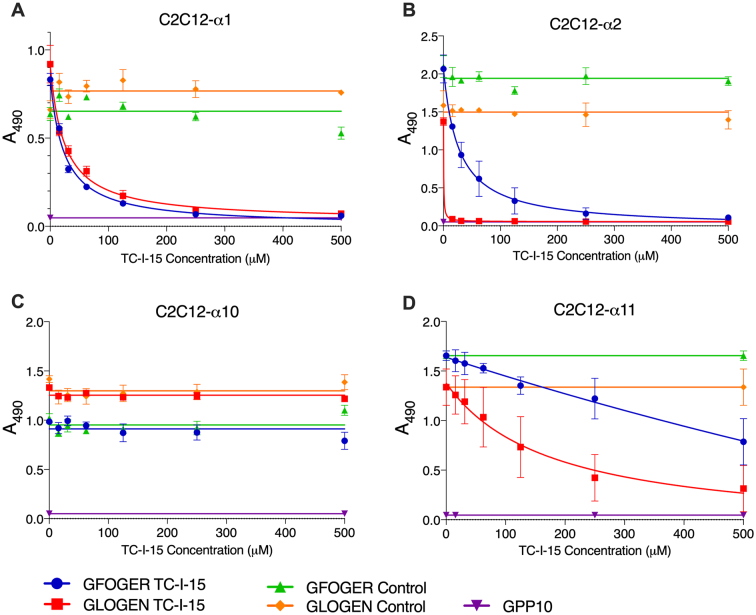
Dose-dependent inhibition of integrin-mediated adhesion of C2C12 cells by TC-I-15. C2C12 cells stably expressing either A, α1β1, B, α2β1, C, α10β1 or D, α11β1 were tested for adhesion to GFOGER (blue) and GLOGEN (red) peptides as described in Materials and methods. NaOH (maximum concentration of 550 μM) was included as a vehicle control on GFOGER (green) and GLOGEN (orange). Adhesion to GPP10 in the absence of inhibitor or vehicle control is shown as the baseline (purple). Adhesion is shown as Mean A_490_ ± SD. Each condition was performed in triplicate and repeated three times. (For interpretation of the references to colour in this figure legend, the reader is referred to the web version of this article.)

Next, to test the effects of these inhibitors on the full-length integrin receptors, C2C12 cells that have been stably transfected to express one of the four integrins were used in static adhesion assays. Plates were coated with GFOGER, GLOGEN or GPP10 (as the negative control) and inhibition dose curves for TC-I-15 and Obtustatin were carried out. Fig. 2 shows the dose curves for TC-I-15-mediated inhibition of the adhesion of C2C12 cells expressing α1β1, α2β1, α10β1 or α11β1 (Fig. 2A-2D respectively). Non-linear regression was used to analyse the curves, as described in Materials and Methods. Firstly, TC-I-15 inhibited α2β1-expressing C2C12 cell adhesion in dose-dependent manner, as anticipated (for GFOGER, IC_50_ = 26.8 μM, R^2^ = 0.9066, *P* < 0.0001 and for GLOGEN IC_50_ = 0.4 μM, R^2^ = 0.9943, *P* < 0.001), but it also inhibited α1β1 (for GFOGER, IC_50_ = 23.6 μM, R^2^ = 0.98, P < 0.001 and for GLOGEN IC_50_ = 24.4 μM, R^2^ = 0.94, P < 0.001). At higher concentrations, α11β1 was also inhibited (for GFOGER, IC_50_ = 3177 μM, R^2^ = 0.80, P < 0.001 and for GLOGEN IC_50_ = 177 μM, R^2^ = 0.70, P < 0.001). For TC-I-15-mediated inhibition of α10β1, and all the control conditions, the preferred statistical model was a horizontal line, i.e., no inhibition was seen. Secondly, the degree of inhibition was peptide-dependent for α2β1 and α11β1, but not for α1β1. For α2β1, complete inhibition of adhesion was seen at very low concentrations of TC-I-15 for the lower-affinity peptide, GLOGEN, but a much higher concentration of TC-I-15 was needed to achieve the same effect for adhesion to GFOGER. A similar substrate-dependence was seen with α11β1. In contrast, for α1β1 the potency was the same for both peptides tested and the IC_50_ values are very similar.

### 3.3 In C2C12 cells, obtustatin inhibits α1β1 only

**Fig. 3:**
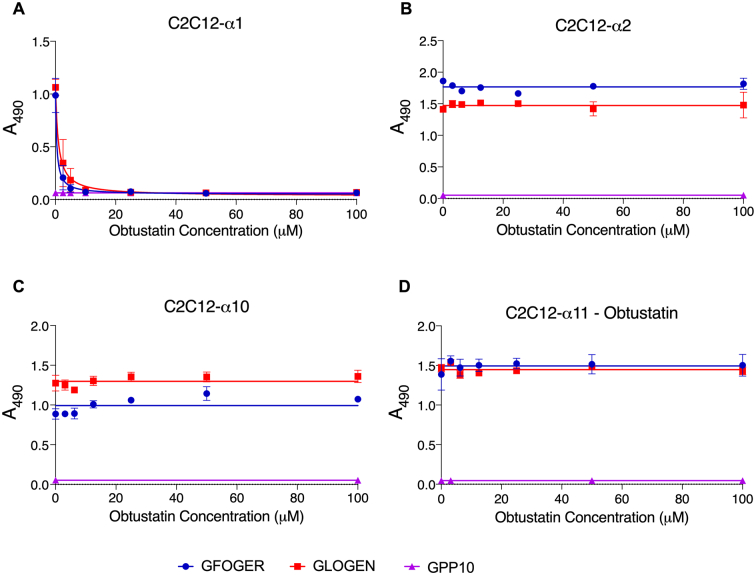
Dose-dependent inhibition of C2C12 cell adhesion to peptides by obtustatin. Cells expressed either A, α1β1, B, α2β1, C, α10β1 or D, α11β1. Adhesion to GFOGER (blue) and GLOGEN (red) is shown, or to GPP10 in the absence of inhibitor or vehicle control (purple). Assays were performed as described in Materials and methods, and results are shown as Mean A_490_, ± SD. Each condition was performed in triplicate and repeated three times. (For interpretation of the references to colour in this figure legend, the reader is referred to the web version of this article.)

The inhibition dose curve assays were repeated with obtustatin (Fig. 3) and non-linear regression was used to analyse the curves as above. Here, obtustatin caused potent inhibition of the adhesion of α1β1 (Fig. 3A) to GFOGER (IC_50_ = 0.45 μM, R^2^ = 0.8920, *P* < 0.0001) and GLOGEN (IC_50_ = 0.96 μM, R^2^ = 0.8496, P < 0.0001). The difference between inhibition on the two peptides was not significant. For the other transfected cells (Fig. 3B-D), no inhibition was observed. This suggests obtustatin is an efficient and specific inhibitor of full length α1β1.

### 3.4 Adhesion of HT1080s is inhibited by TC-I-15 but not obtustatin

**Fig. 4:**
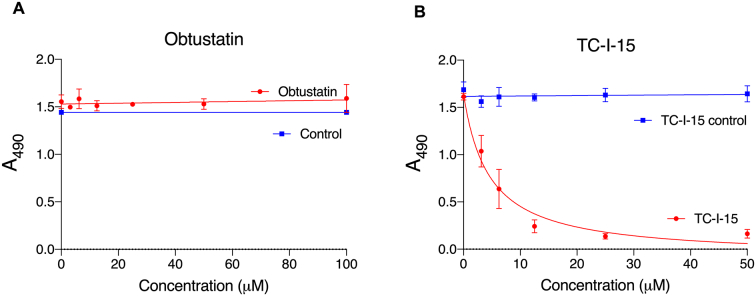
Dose-dependence of inhibition of HT1080 cell adhesion to GFOGER. Assays were performed as described in Material and methods; each inhibitor is shown in red and control curves in blue. The inhibitors under test were A, Obtustatin and B, TC-I-15. Adhesion is shown as Mean A_490_ ± SD. Each condition was performed in triplicate and repeated three times. (For interpretation of the references to colour in this figure legend, the reader is referred to the web version of this article.)

To confirm these findings in a second cell type, inhibition experiments were repeated by measuring the adhesion of HT1080 cells, which express α2β1 but not α1β1 (Ruggiero et al., 1996), to GFOGER (Fig. 4). Obtustatin had no effect on HT1080 adhesion (Fig. 4A). Notably, TC-I-15 inhibited the adhesion of HT1080 cells to GFOGER, *P* < 0.0001 compared to vehicle control, and at lower concentrations than adhesion of C2C12-α2 cells, with IC_50_ 4.53 μM and 26.77 μM, respectively (Fig. 4B). HT1080 is a human fibrosarcoma line, and so expresses both the human α- and β-subunits, whereas C2C12 cells are mouse myofibroblasts, stably transfected with the human α-subunit that dimerises with the mouse β-subunit. Possibly TC-I-15 may have lower affinity for the mouse β-subunit, or the human-mouse heterodimer may communicate imperfectly. HT1080 cells may express lower levels of α2β1, leading to lower avidity of binding to the peptide-coated surfaces. Any of these effects could explain the differences in IC_50_ values for TC-I-15 inhibition shown here.

### 3.5 Full-length, recombinant integrin α3β1 is not affected by TC-I-15

**Fig. 5:**
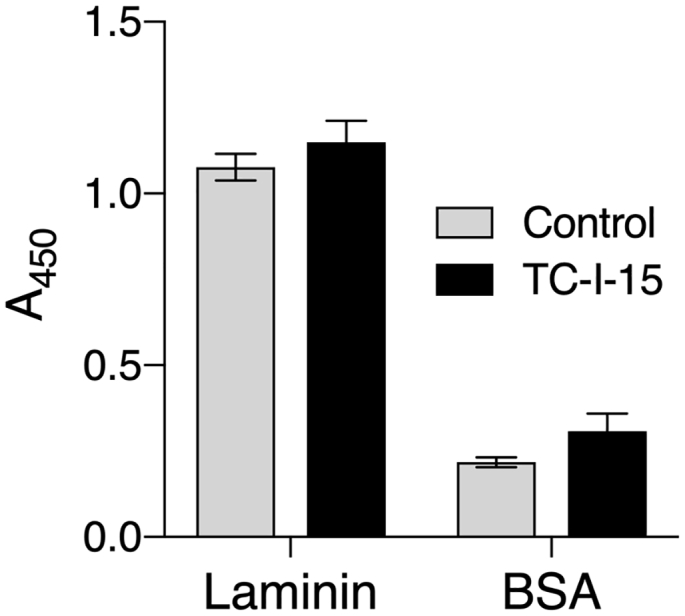
Static adhesion assay showing inhibition by TC-I-15 of full-length isolated α3β1 adhesion to placental laminin-511 or BSA. TC-I-15 (200 μM) is shown in black and the vehicle control data in light grey. Adhesion was quantified by an ELISA detecting the β1 subunit as described in Materials and methods, and is shown as Mean A_450_ ± SD. Data were compared using 2-way ANOVA with Sidak's multiple comparison test, and differences between treatments were not significant. Each condition was performed in triplicate and repeated 3 times.

Here, TC-I-15 has been shown to have broader specificity of inhibition of β1 integrins than reported to date. Since several other integrins contain the β1 subunit (the laminin-binding integrins, α3β1, α6β1, and α7β1, the RGD-binding integrins, α5β1 and α8β1, and the leukocyte integrins, α4β1 and α9β1), we considered that TC-I-15 might also inhibit other β1 integrins. As a representative example of such receptors, adhesion of full-length recombinant human α3β1 was tested with TC-I-15, using laminin 511 as the substrate (Fig. 5). In these conditions, TC-I-15 showed no effect on α3β1.

## 4 Discussion

Currently, TC-I-15 is available commercially as an inhibitor for α2β1. Our results show that TC-I-15 is not specific, but has a broader specificity including α1β1, α2β1 and, at a much higher concentration, α11β1. In contrast, TC-I-15 has, at best, slight effect on the adhesion of α10β1 to GFOGER or GLOGEN peptides. The inhibition is substrate-dependent for α2β1 and α11β1, in that TC-I-15 inhibits adhesion to the lower-affinity GLOGEN at a much lower concentration than applies to GFOGER. The difference in potency of TC-I-15 inhibition of adhesion to these two ligands suggests a competition, where inhibition of adhesion to the higher-affinity GFOGER requires a higher concentration of TC-I-15 than inhibition of binding to GLOGEN. This suggests a reciprocal relationship between GFOGER binding to the upper surface of the αI-domain and TC-I-15 recognition of its inhibitory site on the βI-domain. In contrast, the lower affinity GLOGEN does not require such a high concentration of TC-I-15 to achieve the same effect. Interestingly, this difference in potency is not seen for α1β1, where both peptides are inhibited by similar concentrations of TC-I-15, presumably reflecting the similar affinity of α1β1 for GLOGEN.

Crystal structures provide clear insight into the movement of helix 7 and the C-helix in the α2 I-domain upon binding of GFOGER (Emsley et al., 2000)), and the ligation of E336 at the foot of helix 7 by the β-subunit MIDAS (Carafoli et al., 2013). The location of TC-I-15 binding close to the α2–β1 interface is also well described (Miller et al., 2009), and its effect is understood as allosteric, stabilising the inactive conformation of the integrin. The apparent competition between GFOGER or GLOGEN and TC-I-15 is, however, consistent with this allosteric model. Competition occurs between αI E336 and TC-I-15 at the βI-domain MIDAS, while the role of the collagen peptide is to drive αI helix 7 downwards closer to the βI MIDAS. In activated platelets, the affinity of α2β1 for weaker ligands increases, whereas binding of the high affinity GFOGER is less dependent upon cellular stimulation (Siljander et al., 2004). Thus, in resting cells, GFOGER can provide sufficient binding energy to trigger the reorganisation of the α-subunit MIDAS, C-helix and helix 7 without inside-out integrin activation. This process must include the disruption of the E318 salt bridge, allowing helix 7 to translocate downwards towards the βI-domain MIDAS. A weaker ligand for α2β1, in this case GLOGEN, is unable to provide the same binding energy, and TC-I-15 is more readily able to inhibit α2β1 by blockade of the helix 7–β MIDAS interaction. Hence, helix 7 couples ligand binding at the α-subunit MIDAS to competitive inhibition by TC-I-15 at the β-subunit MIDAS.

The question of selectivity of both TC-I-15 and obtustatin for the different collagen-binding integrins is more difficult to explain. We assume that both inhibitors must interact with both the βI-domain MIDAS and part of the α-subunit at the α–β interface. There is no crystal structure of an intact integrin that contains an αI-domain, and although this interface has been modelled (Miller et al., 2009), no authentic data exists and the relationship between the αI- and βI-domains is not clear. Inspection of the four αI-domain sequences shows several differences in helices 1, 6 and 7, the region of the α subunit most likely to contribute to the footprint of the inhibitors upon these integrins. These may contribute to the lower affinity of TCI-15 for α10 and α11 and for the very high selectivity of obtustatin.

We show here, through the use of recombinant αI-domains, that obtustatin does not compete directly with collagen at the αI-domain. Instead, obtustatin may interact with α1β1 in a similar way to other disintegrins, by binding to the βI-domain at or close to the α–β interface. The action of obtustatin could be analogous to that of TC-I-15, by stabilising both the inactive conformation of the βI-domain and blocking the reorganisation of helix 7 and the C-helix. Structural work is needed to clarify how the selectivity and potency of the active KTSL motif is achieved.

Given that TC-I-15 interacts primarily with the β1 subunit to stabilise the inactive conformation of α1β1 and α2β1, we considered whether TC-I-15 might also inhibit other β1 integrins that lack an αI-domain. The previous report (Miller et al., 2009) had shown no effect of TC-I-15 on the adhesion of platelets, which express α5β1 and α6β1, to the RGD-containing ligands, fibronectin and fibrinogen. Here, we verify this specificity by showing that TC-I-15 had no effect on binding of full-length purified α3β1 to the LDV-containing laminin-511, confirming that TC-I-15 is not a universal inhibitor of β1 integrins. This reinforces the concept that the TC-I-15 interaction involves both the β subunit and the αI-domain. However, the lack of effect on integrin α10β1, which also contains an αI-domain, suggests that the presence of both the β1 subunit and an αI-domain is not sufficient for inhibition to occur. The molecular detail of the interaction remains to be resolved.

In conclusion, it is imperative that integrin inhibitors are thoroughly tested for cross-reactivity before any therapeutic or research use. Obtustatin, in these conditions, was a potent and specific inhibitor for α1β1 adhesion to collagen peptides. The data presented here supports the use of obtustatin for the specific inhibition of α1β1 in cell-based assays, in-vivo assays or potential therapeutic settings. However, because TC-I-15 inhibits α1β1, α2β1 and, at high concentration, α11β1, it must be used with caution in cellular environments where more than one of these integrins is present. TC-I-15 would be a useful tool when inhibition of both α1β1 and α2β1 is required, or where only one of these receptors is present. For example, TC-I-15 been proposed as a potential antithrombotic agent and has been used to block α2β1 on the surface of platelets to inhibit collagen-stimulated platelet aggregation (Miller et al., 2009). Platelets do not express α1β1 or α11β1 and so TC-I-15 would work well here as a specific inhibitor of α2β1. However, in cell-based assays or in-vivo experiments, where more than one of the integrins α2β1, α1β1 and α11β1 are present, such as fibroblasts, endothelial cells or circulating cells such as leukocytes, the cross reactivity of TC-I-15 should be considered.

## Declaration of Competing Interest

RWF is Chief Scientific Officer, CambCol Laboratories, Ely, Cambs, UK.
